# The Latin American Social Medicine database

**DOI:** 10.1186/1471-2458-4-69

**Published:** 2004-12-31

**Authors:** Jonathan D Eldredge, Howard Waitzkin, Holly S Buchanan, Janis Teal, Celia Iriart, Kevin Wiley, Jonathan Tregear

**Affiliations:** 1 The University of New Mexico Health Sciences Library and Informatics Center, MSC09 5100, 1 University of New Mexico, Albuquerque, New Mexico 87131-0001, USA; 2 The University of New Mexico Department of Family & Community Medicine, MSC09 5040, 1 University of New Mexico, Albuquerque, New Mexico 87131-0001, USA

## Abstract

**Background:**

Public health practitioners and researchers for many years have been attempting to understand more clearly the links between social conditions and the health of populations. Until recently, most public health professionals in English-speaking countries were unaware that their colleagues in Latin America had developed an entire field of inquiry and practice devoted to making these links more clearly understood. The Latin American Social Medicine (LASM) database finally bridges this previous gap.

**Description:**

This public health informatics case study describes the key features of a unique information resource intended to improve access to LASM literature and to augment understanding about the social determinants of health. This case study includes both quantitative and qualitative evaluation data. Currently the LASM database at The University of New Mexico  brings important information, originally known mostly within professional networks located in Latin American countries to public health professionals worldwide via the Internet. The LASM database uses Spanish, Portuguese, and English language trilingual, structured abstracts to summarize classic and contemporary works.

**Conclusion:**

This database provides helpful information for public health professionals on the social determinants of health and expands access to LASM.

## Background

Public health practitioners have long recognized the connections between patients' socioeconomic conditions and their health [1-8]. Yet these practitioners and their empirically oriented researcher colleagues have faced difficulties in establishing the precise linkages between socioeconomic variables and sub-optimal health status. Social medicine is a diverse field that studies these relationships between society (and its socioeconomic conditions) and the health of populations. In Latin America, social medicine consists of a widely respected and influential field of research, teaching and professional practice [9]. Professionals working in this field seek to identify and to understand better the linkages between socioeconomic conditions and patients' health.

Until recently, however, most of the knowledge base in this discipline has remained largely unknown outside Latin America. Language barriers and disincentives to distribute this information more widely are two major reasons for this lack of awareness. Some readers might have first learned about Latin American social medicine (LASM) through recent critical reviews [9] or through a special issue of the *American Journal of Public Health *that focused on LASM [10,11].

LASM traces its historic origins to European researchers such as Rudolf Virchow and the belief systems of indigenous cultures. Both the European and indigenous sources of current social medicine practices emphasized the importance of linking social conditions to health status. Contemporary social medicine in Latin America continues to emphasize these linkages between social conditions and the health of populations. Social medicine professionals participate in a wide array of settings in Latin America and represent diverse specialties. Their integration into healthcare systems has varied by era and country [12].

## Construction and content

Innovative approaches to disseminating work in LASM have become increasingly available due to Internet technology. The project "Enhanced Access for Latin American Social Medicine" at The University of New Mexico with funding from the U.S. National Library of Medicine seeks to make information on the connections between social conditions and health problems available to a wide audience. The project has sought to bridge the prior information gap primarily through delivering structured abstracts of social medicine publications in Spanish, English, and Portuguese via the Internet on the LASM database beginning in 2001. Other goals of this project include: publishing full text social medicine electronic journals on behalf of medical societies in Latin America; and, maintaining a repository for key classic and contemporary social medicine publications.

Structured abstracts are posted in three languages in the LASM database at The University of New Mexico for both the classic and contemporary social medicine literatures. The first phase of this project involved preparing and posting the structured abstracts of 25 landmark books, 50 book chapters, and 100 journal articles from the classic social medicine literature in Spanish, Portuguese, and English.

A peer selection committee identified and agreed upon the specific selections of classic books, book chapters, and journal articles to be abstracted for this project. Table 1 lists the members of this committee, representing institutions in Brazil, Chile, Colombia, Cuba, Ecuador, Mexico, the United States, and Venezuela [13]. Representative examples of some of the classic books [14-16], book chapters [17-19], and articles [20-22] can be found in the list of references following this article.

**Table 1:** Members of the Peer Selection Committee

**Country**	**Name**	**Institution**
Brazil	Emerson Elias Merhy	University of Campinas, São Paulo
Chile	Alfredo Estrada L.	Investigation and Training Group in Social Medicine, Santiago
Colombia	Saul Franco Agudelo	National School of Public Health, Bogotá
Cuba	Francisco Rojas Ochoa	National School of Public Health, Havana
Ecuador	Jaime Breilh	Health & Research Advisory Center, Quito
Mexico	Ángeles Garduño	Autonomous Metropolitan University-Xochimilco, Mexico City
Mexico	Asa Cristina Laurell	Secretariat of Health, Mexico City
Mexico	Francisco Mercado Martínez	University of Guadalajara, Guadalajara
Peru	Marcos Cueto	Peruvian University "Cayetano Heredia," Lima
United States	Elizabeth Fee	National Library of Medicine, Bethesda, MD
United States	Norman Frankel	American Medical Association, Chicago, IL
United States	Allen Jones	American Public Health Association, Washington, DC
United States	Antonio Ugalde	University of Texas, Austin, TX
Venezuela	Oscar Feo	Department of Public Health, Maracay
Venezuela	Maria Urbaneja	Latin American Social Medicine Association and Foreign Ministry of the Venezuelan national government, Caracas

The contemporary literature summarized in the LASM database has been drawn primarily from the 12 journals currently or previously published in Latin America. Table 2 lists the 12 journals, with their titles translated into English. These specific journals also have been identified and approved by the peer selection committee. The website that hosts the LASM database provides further information about this committee's members, including their institutional affiliations and areas of research. The peer selection committee consists of experts in social medicine and information technology. The LASM steering committee based at The University of New Mexico meets with the peer selection committee twice a year via online conferencing to decide on selection policies, actual lists of resources slated for inclusion in the LASM database, and administrative matters regarding the project [23].

**Table 2:** Current journal subscriptions monitored for noteworthy articles on social medicine

**Country**	**Title**
Argentina	Cuadernos Médico Sociales (Medico-Social Notebooks) 197?-
Argentina	Salud Problema y Debate (Health – Problem and Debate) +
Brazil	*Cadernos de Saúde Pública (Notebooks of Public Health) 1985-
Brazil	Ciência & Saúde Coletiva (Science and Collective Health) 1996-
Brazil	Interface (Interface) 1997-
Brazil	Revista Brasileira de Epidemiología (Brazilian Journal of Epidemiology) 1998-
Brazil	Saúde e Sociedade (Health and Society) 1992-
Brazil	Saúde em Debate (Health in Debate) 1976-
Cuba	Archivos del Ateneo Juan César García (Archives of the Juan César García Circle) 2000-
Cuba	*Revista Cubana de Medicina Tropical (Cuban Journal of Tropical Medicine) 1996-
Cuba	Revista Cubana de Salud Pública (Cuban Journal of Public Health) 1975-
Mexico	Salud Problema (Health – Problem) +

Notes: * Indexed by the MEDLINE database from the National Library of Medicine + Start date occurred during 1900s but exact date not known

As a pilot, the project has made two social medicine journals available in electronic full text format. Several issues of these journals, *Saúde em Debate *(Brazil) and *SaluCo Bulletin *(Cuba), are available from the host website. The host website also posts structured abstracts of important articles from these full text journals.

The LASM database encompasses several broad themes within LASM. Subject areas include the history, theories, methodologies, and organizational dimensions of social medicine. Other subjects pertain to institutional analysis, social/critical epidemiology, and strategic planning. Table 3 summarizes the specific topics emanating from these broad subject areas. Readers will recognize that many of the subjects have direct bearing on the health of populations, such as health disparities and managed care.

**Table 3:** Subjects covered by Latin American social medicine

Health policy analysis	Violence and health
Medical education reform	Mental health services and mental health policies
Primary care research and preventive services	Determinants of mental illness in race, ethnicity, social class, or gender
Strategic planning	Indigenous, complementary and herbal medicine
Environmental health	Social, environmental and nutritional causes of infant and perinatal mortality
Labor and health	Economic development, demographic change, and aging
Ethnic/racial disparities and health	Socioeconomic barriers to cancer prevention
Social class disparities and health	Social processes of alcohol and drug abuse
Gender disparities and health	Chronic illnesses
Infant and perinatal mortality	Urban health
International health	Managed care

The LASM database is a web application developed using the Cold Fusion application server. The data are stored in a Microsoft SQLServer relational database. The data sources are the original Latin American publications, which are summarized in Spanish, Portuguese, and English languages in structured abstract format. The English-language structured abstracts are quality checked by the principal investigator, who reviews the abstracts for substantive content, and then by the librarian investigator who reviews the structured abstracts for final quality assurance purposes. Each record contains fields for author, title (book, book chapter, or article), place of publication, publisher and structured abstracts in each language. Records contain volume, number, and pagination when applicable. All records contain terms the from Medical Subject Headings (MeSH) system, a controlled vocabulary developed and maintained by the National Library of Medicine. Users can search the database on controlled fields of author, title, and MeSH terms in any of the three available languages. Full text abstract searching is also available. In addition to the abstract searching facilities, the LASM database can be browsed alphabetically by title. The browsing interface offers a convenient way to become familiar with the extent and diversity of the LASM literature.

## Utility and discussion

From January 2002 through December 2003, a total of 17, 853 visits were made to the website hosting the LASM database. The largest numbers of visits in descending rank order originated from Brazil, Mexico, Argentina, Spain, and Colombia. A preliminary, qualitative evaluation has been favorable. Following completion of this project, we will conduct and publish a comprehensive summative evaluation. A total of 250 structured abstracts in Spanish, Portuguese, and English had been posted to the LASM database as of June 30, 2004. The host website presents more detailed information about this project, as well as the structured abstracts themselves.

The LASM database comprises a dynamic and searchable web application containing structured abstracts in English, Portuguese, and Spanish. It is designed using industry standard web application design principles, but its content is unique to the LASM domain.

A database searching design and utility problem unique to the LASM database, and other web applications like it, relates to the problem of web-based multilingual searching. Widely available search engines cannot preprocess a multilingual language translation of search terms (e.g. retrieving results containing the Spanish word "pública" for the English search term "public"). Additionally, search engines do not recognize that the unaccented "publica" (as it might be entered by an English speaker as a search string) might be the same word as the Spanish accented "pública" and therefore will not return the user's expected search result. Key combinations and modifiers that are used to create special and accented characters in word processing programs like Microsoft Word often do not work in browser based search and form fields. Our primary instruction to users for constructing search terms containing special and accented characters (i.e., diacritics) suggests that they use a separate text editor that accepts keyboard modifiers to construct special and accented characters to build the search term and then copy and paste the completed search string into the field. On our search pages we also list common special and accented characters in Spanish and Portuguese that users can copy and paste into browser based search fields.

Although there are no other alternatives for entering special and accented characters into browser form fields, both methodologies are somewhat cumbersome. Therefore, to improve the searching utility of the LASM database we also preprocess entered search strings to allow the search engine to perform selected character substitution on the entered search string and attempt to solve the accented character problem from the search engine side. All search strings are first passed through a regular expression routine, which substitutes single character search engine wildcards for the following characters: A, E, I, O, U, a, e, i, o, u, N, C, n, c. This effectively removes potential special and accented character misspellings. To return to the previous example, a user might enter the search string "publica" ("public" in English) in a search field as an attempt to find article titles that contain the Spanish or Portuguese word "pública". Properly spelled, "Pública" uses the accented character "ú" rather than "u" (ASCII 163 rather than ASCII 117). Unfortunately, current search engines are not intelligent enough to infer that "pública" is a match for the search string "publica". Therefore, the literal search for publica (unaccented) will not return any search results that contain "pública" although there are hundreds of instances of the word "pública" in the LASM database.

In the case of "publica", the preprocessed search string that is finally submitted to the search engine is "p*bl***". The search engine will return all seven-letter word matches that contain the letters p, b, and l in the first, third, and fourth positions respectively. The net effect of this technique is to under-specify the search result. That is, the search engine may possibly return records that contain other words that happen coincidentally to match the submitted search string. On the other hand, the returned result set can be guaranteed to contain the desired search result.

Problems created from under-specifying the search are limited based on experience gained from using this technique. The positional constraints of submitted characters within the search string generally are restrictive enough to prevent most problems. Specifically, within a limited domain search surface like the LASM database, the likelihood of the occurrence of most alternative word matches is very low.

The character substitution methodology described here is not perfect, and many other alternative strategies for addressing the multilingual search problem have been explored by LASM technical staff. Most of the alternative strategies considered, however, involved much higher costs in terms of acquiring or developing specialized search engine capabilities or much higher abstract preparation costs. Therefore, we chose the character substitution strategy as a compromise between implementation cost and search utility.

## Conclusions

Internet technology via websites and web browsers has created numerous opportunities for public health colleagues to inform one another and to collaborate across wide geographic space. The LASM database clearly demonstrates the efficacy of the Internet for communicating its informative structured abstracts posted in the Spanish, Portuguese, and English languages. This database provides useful information that would otherwise be unavailable for public health professionals on the social determinants of health. Furthermore, it expands access to LASM through its inclusion of both the classic and contemporary literature.

## Availability and requirements

Anyone with access to the World Wide Web and a web browser can access all structured abstracts in the LASM database . As the data reported above indicate, the LASM database already has been accessed steadily since its initial small-scale publicity began in 2002. We hope that public health readers will utilize the LASM database to improve their research, teaching, and practice.

## Competing interests

Neither the authors nor any support personnel possess competing interests related to the LASM database or to this article's publication.

## Authors' contributions

JE conceived of and wrote the initial version of this article, served as an early collaborator on and helped design the project, was responsible as an investigator for acquiring journals for the Latin American social medicine collection, provided quality assurance editing on structured abstracts, played a major role in the formative evaluation of the project, and coordinated all revisions to this article. HW initiated and designed the project, served as principal investigator, obtained funding, translated and edited abstracts in the English-language, and edited this manuscript. HSB served as co-principal Investigator, helped design this project, obtained funding, and led the effort for the formative evaluation of this project. JT helped administer the project and edited this manuscript. CI coordinated the project, wrote Spanish-language structured abstracts, and provided reference materials for this article. KW and JT developed all programming aspects of the website including the search strategies, managed the web interface and underlying database and contributed the text for portions of this article.

## Pre-publication history

The pre-publication history for this paper can be accessed here:


